# Outcomes of early post-discharge cardio-geriatric care in frail patients after acute heart failure: a before-and-after study

**DOI:** 10.1186/s12877-025-05883-z

**Published:** 2025-04-09

**Authors:** Chukwuma Okoye, Tessa Mazzarone, Alberto Finazzi, Guarino Daniela, Adriana Antonella Bruni, Lorenzo Maccioni, Giulia Pescatore, Maria Giovanna Bianco, Cinzia Guerrini, Andrea Giusti, Giuseppe Bellelli, Agostino Virdis

**Affiliations:** 1 School of Medicine and Surgery, University of Milano-Bicocca, Via Cadore 48, Monza, 20900 Italy; 2 Fondazione IRCCS San Gerardo Dei Tintori, Monza, Italy; 3 Aging Research Center, Department of Neurobiology, Care Sciences and Society, Karolinska Institutet, Stockholm University, Stockholm, Sweden; 4 Geriatrics Unit, Department of Clinical and Experimental Medicine, University of Pisa, Pisa, Italy

**Keywords:** Heart failure, Follow-up, Outpatients, Outcomes, Cardiogeriatrics

## Abstract

**Background:**

Despite significant advancements in heart failure (HF) management, older adults continue to face poor clinical outcomes. While an integrated, multidisciplinary approach that combines cardiology and geriatric expertise has shown considerable promise, its adoption in practice remains limited. This study aimed to assess whether an early post-discharge Cardio-Geriatric (CG) outpatient service could reduce 1-year mortality compared to usual care (UC), as well as evaluate its impact on 1-year rehospitalization rates and days alive and out of hospital (DAOH).

**Methods:**

In this single-center, controlled before-and-after study, patients aged ≥ 75 years hospitalized for acute HF were included. In the UC group, patients discharged between January 2018 and December 2019 received standard follow-up with referrals to a cardiologist and general practitioner. In the CG group, patients discharged between January 2020 and July 2022 attended CG ambulatory care within three weeks of discharge. Primary outcomes were one-year all-cause mortality, HF readmissions, and DOAH. The impact of CG follow-up was assessed using a 1:1 propensity score matched (PSM) analysis.

**Results:**

A total of 652 patients (mean age 86 years, 56% female) were included in the study, with 477 receiving UC and 175 referred to CG follow-up. Following a 1:1 PSM of 350 patients (50% CG), we observed a significant reduction in 1-year rehospitalizations (36.5% vs. 50.8%, *p* < 0.001) and mortality (20.0% vs. 40.0%, *p* < 0.001) in the CG group. CG patients also had nearly double median DAOH compared to UC patients (300 [IQR: 100] vs. 162 [145] days, *p* < 0.001). Cox regression analysis confirmed that the CG integrated approach was independently associated with a lower risk of mortality [HR 0.34, 95% CI: 0.24–0.47]. Respiratory diseases, neurological conditions, and infections were common causes of readmission.

**Conclusions:**

Early referral to a CG outpatients service post-discharge for acute HF significantly improves outcomes, highlighting the value of integrated care for older adults with complex needs.

**Supplementary Information:**

The online version contains supplementary material available at 10.1186/s12877-025-05883-z.

## Background

Heart failure (HF) is a chronic disabling condition that mainly affects older adults as the result of a complex interplay of age-related diseases and age-associated physiologic changes [1, 2]. Despite substantial improvements in diagnosis, treatment, and management [3], recent data indicate a concerning rise in HF mortality among patients older than 75 [4, 5]. Poor treatment adherence, multimorbidity, and the complexity of managing multiple medications are believed to contribute to these poor outcomes in older patients [6, 7]. Moreover, older patients admitted for acute cardiovascular disease are particularly vulnerable since up to 60% of this population has one or more geriatric syndrome at hospital admission [8], underscoring their heightened vulnerability [9]. Consequently, frailty should be proactively assessed in primary care and clinical settings [10], particularly in HF patients, as recent [11]shows a 2.35-fold higher mortality in frail individuals living in the community compared to their non-frail counterparts.

The use of Comprehensive Geriatric Assessment (CGA) has been shown to be effective in improving survival, reducing risks after hospitalization, and enhancing the functional and cognitive performance of older frail patients. In this regard, recent literature underscores the importance of a multidisciplinary approach to the treatment of HF in the geriatric population [12], emphasizing the value of assessing frailty [1] and evaluating all potential contributors to post-discharge adverse events [13]. As a fact, older adults are at significant risk for both cardiac and non-cardiac events within the first 30 days following discharge, when the risk for adverse outcomes is most pronounced [14]. According to these notions, guidelines and international consensus emphasize the importance of timely outpatient follow-up, ideally within 7 to 14 days, and no later than 28 days post-discharge [3, 15]. However, the integration of cardiology and geriatric care for patients with cardiovascular diseases remains underutilized to date [16]. Moreover, enhancing early post-discharge and long-term outcomes through optimized pre- and post-discharge management of patients with acute HF remains a critical unmet need [15].

In 2020, the tertiary care center of Pisa, established a Cardiogeriatric (CG) outpatient service, aiming to implement best practice standards by tailoring a CGA-based, person-centered approach for frail patients older than 75 years who were recently discharged with acute heart failure from a geriatric unit.

The primary objective of this study is to evaluate whether the establishment of a CG outpatient service reduces one-year mortality compared to usual care; additionally, the study aims to compare the one-year incidence of re-admission, days alive out of hospital (DAOH) until the first re-hospitalization, and to delineate the specific causes of cardiac and non-cardiac re-hospitalizations between the CG and UC groups.

## Methods

This is a controlled before-and-after study, consisting of both retrospective and prospective components. The retrospective component (i.e., control group) of the study included patients discharged from a geriatric acute unit who received usual post-discharge care. The prospective component (experimental group) included outpatients managed by a CG ambulatory service.

### The “before” part: post-discharge usual care

The control group included, with no exclusion criteria, all patients aged 75 or older who were discharged from the geriatric unit of a tertiary care hospital (Azienda Ospedaliero-Universitaria Pisana, Pisa, Italy) with a diagnosis of acute decompensated HF (428.0, 428.2, 428.21, 428.23, 428.31, 428.33, and 428.41 codes of the International Classification of Disease, Ninth Revision Clinical Modification [ICD- 9-CM]) between January 1, 2018, and December 31, 2019. Those with LVEF < 40% were categorized as HFrEF, according to international guidelines. At hospital admission, all patients received a CGA including the Short Portable Mental Status Questionnaire (SPMSQ) [17], the Basic activities of daily living scale (BADL) [18], the Instrumental activities of daily living scale Index (IADL) [19], the Mini Nutritional Assessment Short Form (MNA-SF) [20] (categorized as absent/at-risk or positive) and the Cumulative Illness Rating Scale Comorbidity Index (CIRS-CI) [21]. Frailty was evaluated through the Clinical Frailty Scale (CFS) [22], within 24–48 h, as per the Unit's standardized practice, capturing the patient's baseline frailty prior to hospitalization. Data regarding routinary blood tests, medications and polypharmacy at discharge were also collected. Following hospitalization, patients were referred to either a cardiology outpatient clinic or a general practitioner referral.

### The “after” part: the post-discharge cardio-geriatric ambulatory service

The experimental group comprised patients aged 75 years or older, with a CFS score greater than 3, who attended our CG ambulatory service between January 1, 2020, and July 31, 2022. This selection criterion was based on an institutional healthcare policy, aimed at prioritizing the allocation of specialized geriatric-cardiology resources to patients at higher risk of adverse outcomes due to frailty. In the CG ambulatory service, each patient was evaluated by a HF-specialized internal medicine consultant and a HF-specialized geriatrician (one consultant per day, across two different days each week), supported by two geriatric medicine residents and one nurse. Cardiologists were consulted for cases requiring persistent congestion management or intravenous diuretics in a day hospital setting. The service offered 10 clinic slots per week, with each visit lasting 1 h.

**Fig. 1:**
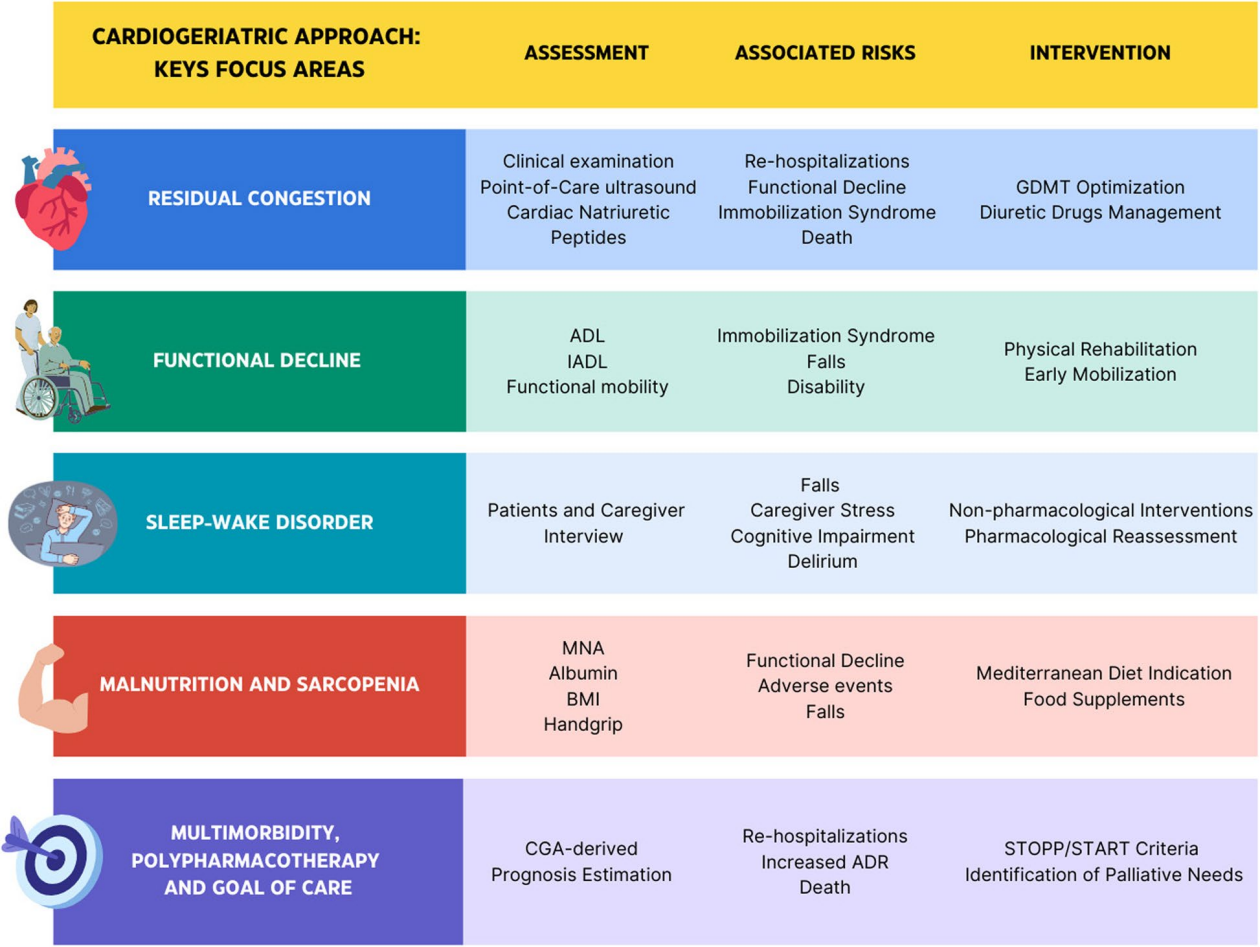
Cardio- Geriatric outpatient service: Key focus areas

Detailed assessments and interventions performed in the CG ambulatory service are outlined in Fig. 1.

Based on the initial CGA and treatment upon discharge, therapeutic strategies, and monitoring were individualized according to clinical guidelines. Patients were re-evaluated within 30 days of hospital discharge, following a structured pre-discharge assessment, according to frailty severity, comorbidity, and BNP levels, as they are independent risk factors for short-term HF re-hospitalization or death [23] (Supplemental Fig. 1). Moreover, we implemented a standardized follow-up protocol for patients, combining CGA with a Point-of-Care ultrasound (POCUS) assessment of systemic congestion severity [24]. Follow-up visits were scheduled according to the physician's clinical judgment, allowing for additional visits as needed.

### Outcomes

The primary outcome was one-year all-cause mortality, secondary outcomes were one-year re-hospitalizations, and DAOH until the first rehospitalization or death. Mortality rates and re-hospitalizations were assessed via phone calls, ambulatory visits, and computerized hospital records. Follow-up data were collected for all patients. For the DAOH calculation, the follow-up period was defined as the interval from the patient’s discharge date to either the date of the first hospital re-admission or death, whichever occurred first. In instances where neither event occurred within the follow-up period, the observation was extended to the end of the study period.

The study adhered to the Declaration of Helsinki and received approval from the Institutional Review Board (IRB, Tuscany Regional Ethics Committee for Clinical Experimentation: FUN-sc 23,956). The IRB waived the need for informed consent for the retrospective phase, while written informed consent was obtained from all participants or their next of kin for the prospective phase.

### Statistical analysis

Statistical analysis was performed using IBM SPSS Statistic (IBM SPSS Statistic version 27.0, IBM Corporation and its licensor 1989–2020) and RStudio (RStudio Team: Integrated Development for R. RStudio, PBC, Boston, MA). Continuous variables were presented as means and standard deviations (SD), ordinal variables as medians and interquartile ranges (IQR), and categorical variables as number of observations and percentages. Mann–Whitney and chi-square tests were used for multiple comparisons. To minimize confounding bias and ensure comparability between treatment and control groups, Propensity Score Matching (PSM) was conducted using the matchit function in R. The matching was performed using a nearest-neighbor algorithm with a 1:1 ratio, based on the following covariates: age, hypertension, sex, BADL, IADL, SPMSQ, CFS, CIRS-CI, HFrEF prevalence and BNP. The balance between the treated and control groups was assessed by comparing standardized mean differences and variance ratios before and after matching. Covariate balance was considered adequate if the standardized mean difference was less than 0.1. For categorical variables, proportions were compared using the chi-square test, while for continuous variables, balance was assessed using t-tests or non-parametric tests as appropriate. Subsequently, mortality risk between the UC and CG groups was evaluated using a Kaplan–Meier estimator. The proportional hazards assumption was checked using Schöenfeld residuals, and the hazard ratio (HR) with a 95% confidence interval (95%CI) was calculated for the CG group compared to the UC group, adjusting for residual confounders identified in the PSM analysis.

## Results

Baseline characteristics are summarized in Table 1. Overall, 652 patients were included in the study, (mean age 86 years, 56% females), showing a high burden of comorbidities (median CIRS – CI: 6, IQR = 3), high prevalence of disability (median ADL 4, IQR = 5) and being mostly frail (median CFS 6, IQR = 4). Four-hundred-seventy-seven individuals were included in the UC group, whereas 175 were followed in the CG service. No differences were found in terms of age or sex distribution between CG and UC patients. Patients in the CG group were less frequently frail, more often independent in ADLs and IADLs, had better cognitive performance, and a reduced burden of comorbidities compared to the UC. Although the prevalence of chronic diseases was similar between the groups, patients in the UC were more frequently malnourished, as suggested by the MNA-SF scores and the mean serum albumin levels. No differences were observed in median ejection fraction (EF) or HFrEF prevalence.

**Table 1:** Comparison between usual care (US) and cardio-geriatric (CG) group

Characteristic	Overall *N* = 652	UC *N* = 477	CG *N* = 175	*p*-value
Sex (M)	287 (44)	210 (44)	77 (44)	0.89
Age (Years)	86 (6)	86 (6)	87 (6)	0.34
ADL	4 (5)	4 (5)	5 (5)	0.006
IADL	2 (5)	2 (4)	2 (5)	0.068
CFS	6 (4)	6 (4)	5 (3)	0.010
CIRS- CI	4 (3)	5 (3)	3 (2)	< 0.001
SPMSQ	2 (5)	3 (5)	2 (4)	0.001
MNA Malnourishment	216 (33.1)	133 (47.4)	83 (27.8)	< 0.001
BNP (mg/dL)	644 (847)	628 (876)	947 (950)	0.012
Creatinine (mg/dl)	1.19 (0.80)	1.19 (0.80)	1.19 (0.74)	0.83
Hemoglobin (g/dL)	11.3 (5.1)	11.2 (5.9)	11.5 (1.9)	0.47
Serum Albumin (g/dL)	3.3 (0.5)	3.2 (0.5)	3.6 (0.3)	< 0.001
Atrial Fibrillation (%)	392 (60.1)	286 (59.9)	106 (60.5)	0.7
Hypertension (%)	442 (67.8)	332 (69.9)	110 (62.8)	0.13
Stroke (%)	83 (12.7)	63 (13.2)	20 (11.4)	0.65
COPD (%)	164 (25.1)	120 (25.1)	44 (25.1)	0.81
CKD (%)	246 (37.7)	181 (37.9)	65 (37.1)	0.76
Ejection Fraction, %	55 (14)	55 (13)	52 (18)	0.36
HFrEF (%)	104 (15.9)	71 (14.8)	33 (18.8)	0.44
CAD (%)	200 (30.6)	170 (35.6)	30 (17.1)	< 0.001
DM2 (%)	202 (30.9)	153 (32.0)	49 (28.0)	0.43
Loop diuretics (%)	638 (97.8)	472 (98.9)	166 (94.8)	0.38
ACE-i (%)	286 (43.8)	190 (39.8)	86 (49.1)	0.24
ARB (%)	98 (14.9)	77 (16.1)	21 (12.0)	0.43
Beta-blockers (%)	515 (78.9)	391 (81.9)	124 (70.1)	0.03
MRA (%)	191 (29.2)	152 (31.8)	39 (22.1)	0.05
ARNI (%)	15 (2.3)	12 (2.5)	3 (2.1)	0.47
SGLT- 2i (%)	16 (2.4)	4 (0.8)	12 (6.4)	0.03

Continuous variables are expressed as mean SD or median with IQR properly

*Abbreviations: UC* Usual Care, *CG* Cardio-Geriatric, *ADL* Activities of Daily Living, *IADL* Instrumental Activities of Daily Living, *BNP* Brain Natriuretic Peptide, *CFS* Clinical Frailty Scale, *SPMSQ* Short Portable Mental Status Questionnaire, *CIRS-CI* Charlson Comorbidity Index, *COPD* Chronic Obstructive Pulmonary Disease, *LVEF* Left Ventricular Ejection Fraction, *ACE-i* Angiotensin-converting-enzyme inhibitors, *ARBs* angiotensin receptor blockers, *ARNI* Angiotensin receptor-neprilysin inhibitor, *SGLT- 2-i* sodium-glucose transport protein 2 inhibitors

Regarding HF medication therapy, patients in the CG group were less frequently prescribed beta-blockers and had higher rates of sodium-glucose cotransporter- 2 inhibitor (SGLT- 2i) use.

At 1-year of follow-up, CG patients had a lower percentage of hospitalizations for all causes (36.5% vs. 52.8%, *p* < 0.001), a lower mortality rate (35% vs. 48.6%, *p* < 0.001), and an increase in DAOH [median DAOH (IQR), 300 (100) vs. 156 (145), *p* < 0.001] (see Table 2).

**Table 2:** Comparison of main outcomes between usual care (US) and cardio-geriatric (CG) group at 1 year of follow-up

	**Overall *N* = 652**	**UC *N* = 477**	**CG *N* = 175**	***p*-value**
Re-hospitalizations (%)	315 (48.3)	252 (52.8)	63 (36.5)	< 0.001
1-year all-cause mortality (%)	270 (41.1)	232 (48.6)	35 (20.0)	< 0.001
DAOH (median, IQR)	174 (145)	156 (145)	300 (100)	< 0.001

*Abbreviations: UC* Usual Care, *CG* Cardio-Geriatric, *DAOH* days alive and out of hospital

### One-year mortality and composite outcome following propensity score matching (PSM)

As shown in Table 3, following the 1:1 PSM, 175 patients receiving UC were compared to 175 patients managed by the CG, with no statistically significant differences observed in main covariates between the groups.

**Table 3:** Balance of covariates before and after propensity score matching

Covariate	Means/Proportion CG (BM, *n* = 175)	Means/Proportion UC (BM, *n* = 175)	Std. Mean Diff. (BM)	Means/Proportion CG (AM, *n* = 175)	Means/Proportion UC (AM, *n* = 175)	Std. Mean Diff. (AM)	Variance Ratio (AM)
Age	86.6529	86.1239	0.0876	86.6529	86.1955	0.0757	0.7640
Male sex	0.5829	0.5451	0.0766	0.5829	0.5771	0.0116	-
Female sex	0.4171	0.4549	− 0.0766	0.4171	0.4229	0.0116	-
ADL	3.6400	3.3082	0.1460	3.6400	3.5657	0.0327	1.0581
IADL	2.9086	2.5304	0.1295	2.9086	2.7600	0.0509	1.0884
SPMSQ	2.9314	3.6541	− 0.2676	2.9314	3.1886	0.0952	0.9382
CFS	4.9200	5.2013	− 0.1710	4.9200	4.9486	0.0174	0.6111
CIRS	3.3314	5.2096	− 1.3008	3.3314	3.4171	0.0594	0.7776
HFrEF	0.2457	0.2222	0.0546	0.2457	0.2629	0.0398	-
BNP	830.0114	895.3941	− 0.0700	830.0114	836.3371	0.0068	1.255

*Abbreviations: UC* Usual Care, *CG* Cardio-Geriatric, *BM* Before Matching, *AM* After Matching, *ADL* Activities of Daily Living, *IADL* Instrumental Activities of Daily Living, *CFS* Clinical Frailty Scale, *SPMSQ* Short Portable Mental Status Questionnaire, *CIRS-CI* Charlson Comorbidity Index, *HFrEF* Heart Failure with reduced Ejection Fraction, *BNP* Brain Natriuretic Peptide

**Fig. 2:**
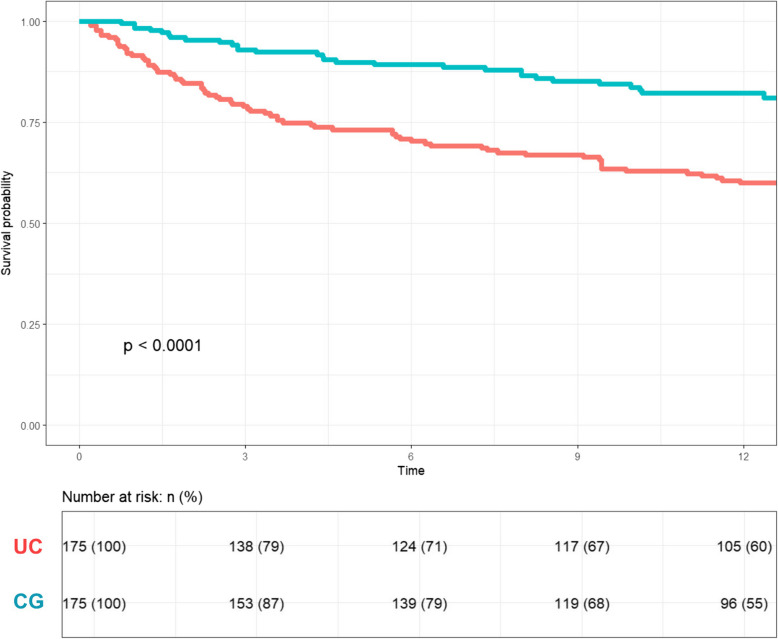
Survival curves in usual care (UC) and cardiogeriatric outpatient service (CG) groups following 1:1 propensity score matching

At 1-year follow-up, CG patients had a significantly lower prevalence of re-hospitalizations compared to those in the UC group (36.5% vs. 50.8%, *p* < 0.001) and a lower mortality (20.0% vs. 40.0%, *p* < 0.001). Patients in the CG group had a 64% risk reduction of the one-year mortality [HR 0.36 (95%CI: 0.26–0.53), log-rank *p* < 0.001)] (see Fig. 2).

Overall, patients managed by CG outpatients service had almost the double DAOH (300 ± 100 vs 162 ± 145 days, *p* < 0.001) compared to UC.

### Causes of re-hospitalization in standard-of-care group and cardio-geriatric group

In the overall cohort, the distribution of readmission causes did not significantly differ between the UC and CG groups. The rate of heart failure relapse (r-HF) was 33.9% in the UC group and 37.5% in the CG group, while readmissions due to other causes (r-OC) were 66% in the UC group and 62.9% in the CG group.

Patients with r-HF exhibited a higher, though not statistically significant, one-year mortality compared to those with r-OC (33.3% vs. 25%, *p* = 0.47). As shown in Table 4, the predominant causes of readmission in both UC and CG groups were respiratory diseases (14.8% vs. 15%), infectious diseases (11.7% vs. 9.3%), and neurological diseases (7% vs. 9.3%). No significant differences in cause-specific readmissions were observed between the CG and UC groups, except for anemia, which was more common in CG patients (4.6% vs. 0.7%).

**Table 4:** Main causes of hospital readmission in 1-year follow-up

**Diagnosis**		**Overall, *N* = 320**	**U**C**, *N* = 256**	**C**G**, *N* = 64**	***p*-value**
**ADHF (%)**		111 (34.6)	87 (33.9)	24 (37.5)	0.59
**Respiratory Diseases (%)**		48 (15)	38 (14.8)	10 (15.6)	0.87
	COPD exacerbation (%)	10 (20.8)	9 (23.6)	1 (10)	
	Pneumonia (%)	18 (37.5)	12 (31.5)	6 (60)	
	Aspiration pneumonia (%)	3 (6.2)	3 (7.8)	0 (0)	
	Other (%)	17 (35.4)	14 (36.8)	3 (30)	
**Infective Diseases (%)**		36 (11.2)	30 (11.7)	6 (9.3)	0.59
	Sepsis (%)	18 (50)	15 (50)	3 (50)	
	UTI (%)	11 (30.5)	9 (30)	2 (33.3)	
	Other (%)	7 (19.4)	6 (20)	1 (16.6)	
**Neurological Diseases (%)**		24 (7.5)	18 (7)	6 (9.3)	0.52
	Ischemic Stroke (%)	14 (58.3)	10 (55.5)	4 (66.6)	
	Delirium (%)	10 (41.6)	8 (44.4)	2 (33.3)	
**CV Diseases (%)**		21 (6.5)	17 (6.6)	4 (6.2)	0.91
**Cancer (%)**		14 (4.3)	11 (4.2)	3 (4.6)	0.89
**AKI (%)**		12 (3.7)	12 (4.6)	0 (0)	0.07
**GI Diseases (%)**		11 (3.4)	9 (3.5)	2 (3.1)	0.87
**Fractures (%)**		8 (2.5)	7 (2.7)	1 (1.5)	0.59
**Electrolyte Imbalances (%)**		5 (1.5)	5 (1.9)	0 (0)	0.26
**Anemia (%)**		5 (1.5)	2 (0.7)	3 (4.6)	0.02
**Other (%)**		25 (7.8)	20 (7.8)	5 (7.8)	1

*Abbrevations: UC* Usual Care population, *CG* Cardio-Geriatric population, *ADHF* Acute Decompensated Heart Failure, *COPD* Chronic Obstructive Pulmonary Disease, *UTI* Urinary Tract Infection, *GI* Gastro-Intestinal Diseases, *CV* Cardio-vascular diseases, *AKI* Acute Kidney Injury

## Discussion

In our quasi-experimental study, we found that early follow-up cardio-geriatric care significantly improved patient outcomes, reducing mortality risk by 66%, markedly lowering both HF-related and all-cause re-hospitalizations, and substantially increasing days alive and out of the hospital (DAOH).

Based on our findings, integrating a tailored cardio-geriatric approach into early post-discharge care could be a critical strategy for improving outcomes in the most vulnerable older patients, emphasizing the need for individualized care plans that address both cardiovascular and geriatric complexities.

Previous studies have shown the efficacy of early post-discharge ambulatory service in reducing adverse events following hospitalization for acute HF [25, 26], but to date, none have specifically dealt with the oldest old individuals, quantifying their frailty and defining a personalized approach based on the domains of the CGA.

Recent data has drawn attention to a concerning mortality trend among individuals aged over 75, diverging from trends observed in younger counterparts [6, 7]. Despite recent international guidelines advocating for comprehensive management of comorbidities and frailty, very few patients are referred to geriatricians.

In our study more than 4 out of 10 individuals died within one-year from hospital discharge, aligning with a previous study on very old patients with HF, describing a 42% one-year all-cause mortality [27]. Interestingly, the significant mortality rate improvement among patients followed by the CG service was particularly pronounced in the early post-hospitalization period. Indeed, within the first 30 days post-discharge, these patients face a high risk of both HF relapse and re-hospitalization for other causes [28, 29]. By integrating BNP levels, comorbidities, and CFS scores at progressive follow-up intervals and tailoring treatment to address specific deficits, the program effectively reduced readmissions. However, the ratio of cardiac to non-cardiac causes of readmission remained unchanged, highlighting the significant impact of non-cardiac conditions. Consistent with prior studies, the most frequent causes of readmission were respiratory diseases, neurological conditions, and infectious diseases [30, 31]. Among modifiable re-admissions, we found a decreased rates in exacerbations of chronic obstructive pulmonary disease (COPD) and aspiration pneumonia, prevalent acute afflictions in frail older patients. In these cases, counseling on proper device handling for COPD, vaccination recommendations, and education of caregivers and patients on appropriate nutrition proved effective in reducing inappropriate hospitalizations.

Non-respiratory infectious diseases were most frequently urinary tract infections, often linked to dehydration and delirium, further compounded by polypharmacy and the use of antihypertensive medications. In this context, a standardized assessment of functional status, nutrition (diet, dysphagia), and therapeutic reconciliation (deprescribing, reassessment of medication adherence) proved effective in reducing adverse events. Finally, we observed reduced rehospitalization rates for acute kidney injury and electrolyte imbalances in patients managed with the CG approach compared to UC. Indeed, electrolyte disturbances and dehydration due to diuretic therapy can lead to complications such as constipation, urinary tract infections, and confusion—conditions that are preventable or treatable through appropriate geriatric assessment, drug dosage reduction [32], and proper caregiver training. More in depth, while our study focused on the impact of early post-discharge cardio-geriatric care, medication optimization, including deprescribing, remains a critical aspect of managing frail older patients with HF [33]. Unfortunately, deprescription data for the standard of care group were not available, preventing direct comparison. Given its relevance in reducing polypharmacy-related adverse outcomes, deprescribing represents an important avenue for future research, further investigations are warranted to define its role within the cardio-geriatric care model.

Crucially, our findings suggest that an integrated CG approach significantly increases DOAH, which may serve as a more meaningful endpoint than mortality or rehospitalization in very old patients with HF. By minimizing unnecessary hospitalizations, this approach may alleviate pressure on the healthcare system, freeing up resources for more acute cases.

The demonstrable benefits of this approach extend beyond clinical outcomes, impacting healthcare utilization and costs. As the global population continues to age, embracing such comprehensive care models becomes imperative for improving the overall health trajectory of older individuals. Future research should explore the generalizability of the CG model across various healthcare settings and diverse populations, assessing its sustainability and cost-effectiveness.

This study is subject to several limitations that warrant consideration. First, potential selection bias may have influenced the outcomes, as patients who were more capable of traveling to the CG outpatient service were more likely to be evaluated, potentially leading to the underrepresentation of patients who were too frail or unable to attend. Furthermore, the CG group had better functional status at baseline, which may suggest additional selection bias. While multiple adjustment and propensity score matching were used to address this issue, unmeasured factors, such as a cohort of patients discharged to palliative care, which may be more prevalent in the UC group, could not be included in the model. This might have contributed to the notably high relative risk reduction observed in the CG group.

Moreover, the balance between the CG and UC groups was disrupted by the COVID- 19 pandemic, which significantly slowed the enrollment process between 2020 and 2021. Despite these challenges, the robustness of the findings is supported by comprehensive adjustments for frailty, multimorbidity, and primary risk factors, facilitated by a 1:1 PSM ratio that helps to mitigate these biases. Importantly, while adverse events were more frequent in the first cohort, only the second cohort was directly affected by COVID- 19, with lockdown measures limiting enrollment and COVID-related mortality potentially underestimating the benefits of the CG approach. Nevertheless, the intervention consistently demonstrated significant improvements in outcomes, underscoring its clinical relevance.

Moreover, the intervention was assessed only in frail and pre-frail patients (CFS > 3), without including a cohort of non-frail individuals, like those receiving UC. However, it should be noted that frail and pre-frail patients are at higher risk for adverse outcomes and are often more complex due to the presence of geriatric syndromes, multimorbidity, and polypharmacy. Therefore, they are the ones who most require a multidisciplinary assessment based on CGA. Notwithstanding, future studies could consider including non-frail patients to assess the broader impact of the CG intervention. Nevertheless, the study's single-center design limits the generalizability of the findings to other settings. Furthermore, the absence of data on post-heart failure rehabilitation could affect the interpretation of the outcomes. As a before-and-after study, there is also the possibility of unmeasured confounders influencing the results, though extensive adjustments for baseline comorbidities support the replicability of the observed effects. Lastly, improvements in outcomes over time might be partly attributable to enhanced clinician performance, underscoring the need for further validation through randomized controlled trials comparing cardiological and geriatric outpatient care across multiple centers.

## Conclusions

The CG ambulatory service led to significant improvements in reducing one-year all-cause mortality, as well as hospital readmissions for both HF and non-HF causes, among frail older patients recently discharged after an acute HF episode. By significantly increasing the DOAH, this integrated approach not only improved clinical outcomes but also enhanced overall patient well-being. These findings underscore the potential of the CG ambulatory model to address the complex needs of the older HF population, emphasizing the importance of a tailored, multidisciplinary care strategy. Further investigations into the scalability and impact of this model across different healthcare settings.

and diverse patient populations are warranted to confirm and expand upon these promising results.

## Supplementary Information

Supplementary Material 1. Supplemental Figure 1. Structured pre-discharge assessment and timing for Cardio-Geriatric outpatient service first visit.

## Data Availability

The datasets used and/or analysed during the current study are available from the corresponding author on reasonable request.

## Abbreviations

ACE-i: Angiotensin-converting-enzyme inhibitors
ADHF: Acute Decompensated Heart Failure
ADL: Activities of Daily Living
AKI: Acute Kidney Injury
ARB: Angiotensin Receptor Blockers
ARNI: Angiotensin Receptor-Neprilysin Inhibitor
BADL: Basic Activities of Daily Living
BNP: Brain Natriuretic Peptide
CFS: Clinical Frailty Scale
CG: Cardio-Geriatric
CGA: Comprehensive Geriatric Assessment
CIRS-CI: Cumulative Illness Rating Scale Comorbidity Index
COPD: Chronic Obstructive Pulmonary Disease
CV: Cardiovascular
DAOH: Days Alive and Out of Hospital
GI: Gastrointestinal
HF: Heart Failure
HFrEF: Heart Failure with Reduced Ejection Fraction
IADL: Instrumental Activities of Daily Living
LVEF: Left Ventricular Ejection Fraction
MNA-SF: Mini Nutritional Assessment Short Form
MRA: Mineralocorticoid Receptor Antagonists
PSM: Propensity Score Matching
SGLT- 2i: Sodium-Glucose Cotransporter- 2 Inhibitors
SPMSQ: Short Portable Mental Status Questionnaire
UC: Usual Care
UTI: Urinary Tract Infection
