# Supplementary figures and images for: Outcomes of early post-discharge cardio-geriatric care in frail patients after acute heart failure: a before-and-after study
Source: BMC Geriatr. 2025 Apr 9;25:236. doi: 10.1186/s12877-025-05883-z (PMC11983887; doi:10.1186/s12877-025-05883-z)

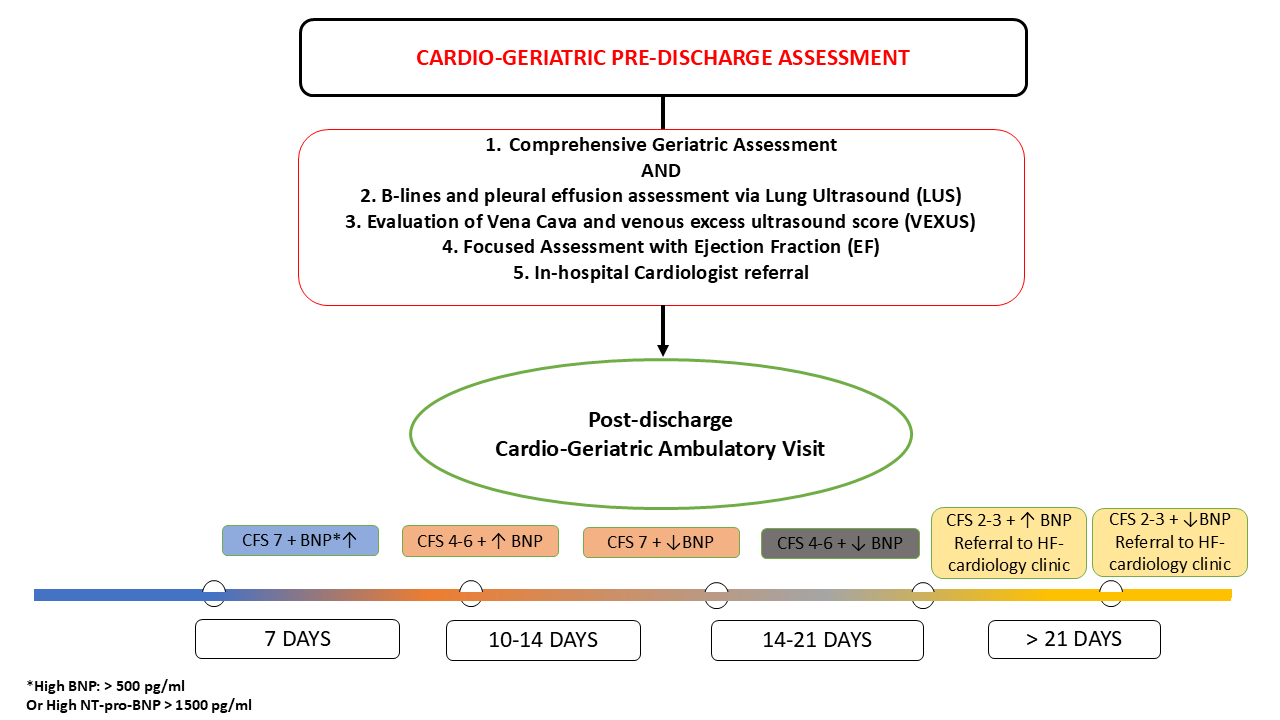

Supplement: Supplementary file 1 — Supplementary Material 1. Supplemental Figure 1. Structured pre-discharge assessment and timing for Cardio-Geriatric outpatient service first visit. [file 12877_2025_5883_MOESM1_ESM.tif]
